# The Effects of *Tachykinin1* Gene Products on Prepubertal Dabry’s Sturgeon (*Acipenser dabrynus*) Pituitary Hormone Secretion and Gene Expression

**DOI:** 10.3390/ani14020227

**Published:** 2024-01-11

**Authors:** Kan Xiao, Hongtao Huang, Xuetao Shi, Tingting Shu, Xu Cheng, Hejun Du, Jing Yang

**Affiliations:** 1 Hubei Key Laboratory of Three Gorges Project for Conservation of Fishes, Yichang 443100, China; xiao_kan@ctg.com.cn (K.X.); huang_hongtao@ctg.com.cn (H.H.); shi_xuetao@ctg.com.cn (X.S.); shu_tingting@ctg.com.cn (T.S.); cheng_xu2@ctg.com.cn (X.C.); du_hejun@ctg.com.cn (H.D.); 2 Chinese Sturgeon Research Institute, China Three Gorges Corporation, Yichang 443100, China

**Keywords:** endangered species, neuropeptide, SP, NKA

## Abstract

**Simple Summary:**

*Tachykinin1* (*tac1*) gene products participate in the regulation of hormone secretion in the pituitary of Dabry’s sturgeon. Two products of *tac1* gene, Substance P (SP) and neurokinin A (NKA), were initially confirmed to regulate the gene expression of two gonadotropins (*lh* and *fsh*) in the Dabry’s sturgeon pituitary tissue by intraperitoneal injection in vivo and co-culture of primary cells in vitro. This research presents the initial investigation into the reproductive role of SP and NKA in Dabry’s sturgeon, offering a valuable insights for advancing efficient ripening techniques in artificial breeding of sturgeon.

**Abstract:**

As an ancient and endangered species unique to the Yangtze River in China, the wild population of the Dabry’s sturgeon has become scarce. Due to the long time till the first sexual maturity of Dabry’s sturgeon, the population of artificially bred Dabry’s sturgeon recovered slowly. As a member of the tachykinin family, TAC1 has been reported to have a variety of functions in mammals such as pain control, smooth muscle contraction and reproductive cycle regulation, but the function of Tac1 in fish has been rarely reported. In this study, we synthesized two *tac1* gene products, Substance P (SP) and neurokinin A (NKA), and further verified the effect of two *tac1* gene products on the secretion of related hormones in the pituitary of Dabry’s Sturgeon by intraperitoneal injection and co-culture of primary cells. Expression studies revealed that the newly cloned *tac1* were mainly distributed in the hypothalamus and pituitary tissue of the brain. In prepubertal Dabry’s sturgeon, this study showed that the two gonadotropins’ mRNA levels in pituitary tissue can be significantly increased by SP and NKA through intraperitoneal injection, and the LH protein level in serum was also increased. Further study showed that both NKA and SP could promote the two gonadotropins’ mRNA expression in pituitary cells of Dabry’s sturgeon. In addition, we explored the optimal dose and time of SP and NKA on pituitary cells is 24 h and over 10 nM. These results, as a whole, suggested that *tac1* gene products play an important role in gonadotropin release and gonadal development in prepubertal Dabry’s sturgeon.

## 1. Introduction

Dabry’s Sturgeon (*Acipenser dabryanus*) is a rare and unique fish mainly distributed in the Yangtze River basin of China. Due to overfishing, pollution and habitat change and destruction, the wild population of Dabry’s sturgeon has declined sharply, and is now endangered [1,2]. Although the technology of artificial breeding of Dabry’s sturgeon has been successful, the population recovery of Dabry’s sturgeon is slow due to the long sexual maturity time and the asynchronous gonad development of male and female fish. The delayed sexual maturity between male and female Dabry’s sturgeons (4–7 years for males and 6–8 years for females) increases the difficulties in their protection compared with that of fishes that have a short-span maturity [3,4]. Therefore, in order to quickly restore the population of Dabry’s sturgeon, it is necessary to find a highly efficient sturgeon ripening agent.

At present, the identification and research of genes related to gonadal development and initiation of sexual maturation in fish are far behind those of higher vertebrates. Fish receive external stimuli through sensory organs and send them to the brain. GnRH is secreted by the hypothalamus to stimulate Gth, which acts on the gonad and regulates the development of fish gonad and the initiation of sexual maturation [5]. As the upstream of GnRH, there are several neuropeptides that regulate GnRH secretion, such as kisspeptin and tachykinin [6]. The tachykinin family (TAC) is a group of short peptides with an α-amideated FXGLM motif at the end of the carbon, with X representing a hydrophobic aromatic residue or a branched adipochain [7]. There are five tachykinins in mammals, including the Tac1 gene Substance P (SP) and neurokinin A (NKA), the Tac3 gene product neurokinin B (NKB), the Tac4 gene products endokinins (EKs), and hemokinin 1 (HK-1). These tachykinins plays the role as endocrine hormones, neurotransmitters, and autocrine/paracrine regulators in mammals [8]. Among them, namely *Tac1* encoding SP and NKA, *Tac3* encoding NKB, and *Tac4* encoding HK-1 or EKs [9], *Tac1* encoding product SP plays an important role in a variety of physiological behaviors such as pain perception [10], neurogenic inflammation [11], smooth muscle contraction [12,13] and obesity regulation [14]. In study of rats, it was found that *Tac1* encoding product NKA could promote the release of prolactin (PRL) [15], while another study showed that SP could directly induce the increase in LH secretion in pigs [16]; these studies, in addition, proved that TAC1 may also involve in reproduction.

Compared with mammals, studies on Tac in fish started relatively late, and mainly focused on the regulation of fish reproduction by *tac3* encoding products (NKB and NKBRP). Recent studies have found that fish *tac3* encoding products could induce the increase in Gth protein secretion levels in zebrafish (*Danio rerio*) [17,18], tilapia (*Reochromis mossambicus*) [19], and goldfish (*Carassius auratus*) [20]. In addition, it was found that *tac3* encoding products could directly induce the synthesis and secretion of prolactin [21] and somatolactin [22] in the pituitary gland of grass carp. So far, there are only few studies have reported the *tac1* gene product SP is involved in zebrafish embryogenesis [23] and grass carp pituitary hormone secretion [24]. To exploring the regulatory effect of *tac1* gene products on pituitary gene expression of Dabry’s sturgeon. We first cloned *tac1* gene and investigated its expression level in various tissues of Dabry’s sturgeon. Then, we synthesized two polypeptide products of *tac1* gene, SP and NKA, and investigated the effect of *tac1;* gene products on the gene expression of Dabry’s sturgeon in vivo and in vitro, respectively, and initially investigated the optimal dose and time of SP and NKA action on pituitary cells of Dabry’s sturgeon.

## 2. Materials and Methods

### 2.1. Experimental Animals Preparation

A group of 18 artificially bred Dabry’s sturgeons, aged 7 months, were randomly selected and temporarily kept in a breeding pool for two weeks. The water quality in the breeding pool was the same as that in a normal artificial breeding pool. These Dabry’s sturgeons had a total length ranging from 45 to 55 cm and weighed between 0.4 and 0.6 kg. All the sturgeons originated from the Chinese Sturgeon Research Institute in Yichang.

### 2.2. In Vivo SP and NKA Treatments and Sampling Procedure

First, based on the *tac1* gene sequence of Dabry’s sturgeon found on the NCBI: https://www.ncbi.nlm.nih.gov/ (accessed on 12 January 2021), the peptide sequences of SP and NKA were identified as PKPHQFFGLM and QKLNSFVGLM, respectively. The peptides SP and NKA were synthesized by Wuhan Dia-an Biotechnology Co., Ltd. (Wuhan, China). Eighteen Dabry’s sturgeons were randomly divided into three groups. The peptides SP and NKA powder were dissolved in 0.7% normal saline to a final concentration of 250 μg/mL, and each group of fish was intraperitoneally injected with either the SP peptide solution, NKA peptide solution or 0.7% normal saline. Samples were collected at 3 h and 24 h after injection. During the sampling process, the sturgeon was anesthetized by 0.75% tricaine methasulphonate (MS222) (Sigma, St. Louis, MO, USA), and blood samples were collected through the tail vein, and the hypothalamus, pituitary gland, liver gonad and other tissues were collected after spinosectomy according to the animal use regulations of Chinese Sturgeon research institute.

### 2.3. Pituitary Cell Culture

The pituitary tissue was extracted and soaked in 75% ethanol solution for 1 min, then washed three times in PBS (Gibco, Waltham, MA, USA) balanced salt solution. The pituitary tissue was cut into 1 mm^3^ tissue blocks with scissors, and the tissue blocks were digested with 0.25% trypsin solution (Gibco, Waltham, MA, USA). After digestion in 37 °C water bath for 10 min, an equal amount of MEM medium solution (Gibco, Waltham, MA, USA) containing 10%FBS (Gibco, Waltham, MA, USA) was added to terminate digestion. Remove the supernatant after centrifugation for 5 min at 4 °C and 1500 rpm. The resulting precipitates were re-suspended with a MEM medium solution containing 10%FBS and 100 IU penicillin and streptomycin and inoculated into 24-well plates that were pre-coated with polylysine. Approximately 2.5 × 10^6^ cells were inoculated in each hole of the 24-well plate.

### 2.4. Total RNA Extraction and Reverse Transcription

For the extraction of total RNA from tissues, the tissue blocks were first broken using a tissue crusher, then Trizol reagent (1 mL/tube) was added (Invitrogen, Carlsbad, CA, USA). For RNA in cultured cells, Trizol reagent (500 µL/well) was added to 24-well culture plates. Total RNA was then extracted and dissolved in RNase-free water following the instruction of RNA Easy Fast Tissue/Cell Kit (Tiangen Biotech Co., Ltd. Beijing, China). After total RNA was extracted and quantified by the NanoDrop One (Thermo Scientific, Rockford, IL, USA), reverse transcription was performed with tm III Strand 1 cDNA synthesis kit (gDNA digester plus) (Yeasen Biotech Co Ltd., Shanghai, China). Then, 500 ng total RNA was taken as the template of a 20 µL system of the cDNA synthesis kit. The cDNA was synthesized following the instructions. The retrotranscriptional product was quantified with NanoDrop One (Thermo Scientific, Rockford, IL, USA), diluted to 50 ng/µL, and used for subsequent Real time-qPCR reaction.

### 2.5. Real-Time Quantitative PCR

The *fsh* and *lh* mRNA expression level was detected by using ABI 7500 real-time PCR system (Applied Biosystems, Carlsbad, CA, USA) with specific primers. The primers of *fsh* and *lh* was designed from their CDS regions, respectively. *β-actin* was used as the internal reference gene (the information of the primers refer to Table 1). The continuous dilution of plasmid DNA (3 µM to 0.375 µM, 1/2 x at each time) containing *fsh* and *lh* coding sequences worked as the standard for real-time PCR for data calibration.

The plasmid DNA for calibration was constructed according to a previous report [25]. The CDS regions of the *lh* and *fsh* gene of Dabry’s sturgeon were searched on NCBI, and the full-length CDS sequences of *lh* and *fsh* were obtained by designing primers based on the CDS sequences (Table 1) and amplification by the 2 × Hieff Canace^®^ PCR Master Mix (Yeasen Biotech Co Ltd., Shanghai, China) with a PCR condition: 98 °C 5 min; 98 °C 10 s; 59 °C 30 s; 72 °C 30 s; a total of 40 cycles. The PCR products of the *lh* and *fsh* CDS sequences were then connected with pcDNA3.1 and cloned.

The qPCR in 20 µL worked with RealUniversal Color PreMix (SYBR Green) (Tiangen Biotech Co., Ltd. Beijing, China) following the instructions, and the volume of the cDNA or plasmid DNA added to the system was 2 µL. The qPCR condition was as follows: 95 °C 15 min; 95 °C 30 s; 58~60 °C 30 s; 72 °C 30 s; a total of 40 cycles. The specificity of the qPCR reaction was verified by melt curve analysis at the end of the reaction.

### 2.6. Detection of Lh Content by ELISA

The Lh protein in serum and in the pituitary cell culture-medium was determined by enzyme-linked immunosorbent assay that has been reported previously [25]. First, the recombinant Lh protein and its monoclonal antibody were synthesized. According to the sequence of Lh polypeptide “LRLCEPVNETISAEKEECPKCLLIQTSICSGSCPTKDPV FKSALSTVQQHVCTYKDVRVTVTLPDCPPGVDPHFTFPLALSCECSLCRMESSDCTIQSVGPSDCMSGELAIQN”, the antibody was synthesized by Wuhan Dia-an Biotechnology Co., Ltd. (Wuhan, China), and the specificity of the antibody was detected via Western blot (Figure S1). The antibody was coated in the Costar 96-well black plate (Thermo Fisher, Waltham, MA, USA) and incubated overnight at 4 °C. After that, each well was washed three times with washing buffer to remove the non-specific binding of the primary antibody. The serum sample or cell culture-medium was added to the well and incubated for 1 h. Similarly, after three times of washing, biotin-labeled antibody was added and incubated for 1 h, followed by three times of cleaning, horseradish peroxidase-labeled avidin (A-HRP) (Thermo Scientific, Rockford, IL, USA) was added and incubated for 45 min, and then washed four times. Next, 0.1 mL TMB substrate application solution was added into each well. After incubation at 37 °C for 10 min, 2 mol/L H_2_SO_4_ 0.05 mL was added to terminate the reaction. Finally, the fluorescence signal was detected by FluoStar optima-fluorescent plate Reader.

## 3. Results

### 3.1. Molecular Cloning of Dabry’s Sturgeon TAC1 and Tissue Experssion

**Figure 1:**
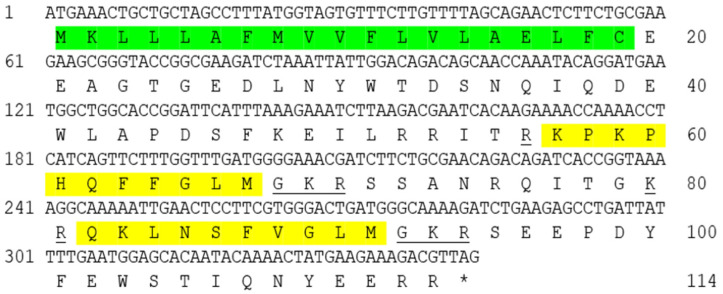
cDNA region of *tac1* from Dabry’s sturgeon. Notes: signal peptides are marked in green; mature peptides are marked in yellow; the cutting sites are underlined.

**Figure 2:**
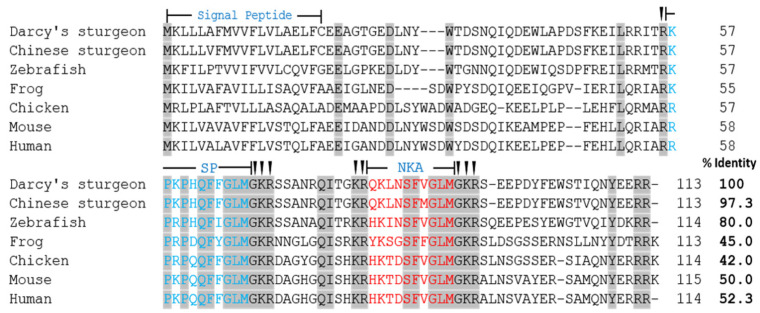
Amino acid sequence alignment of Tac1 from Dabry’s sturgeon and other vertebrates. Notes: conserved amino acids are marked in gray; the amino acid sequences of SP and NKA are marked in blue and red.

**Figure 3:**
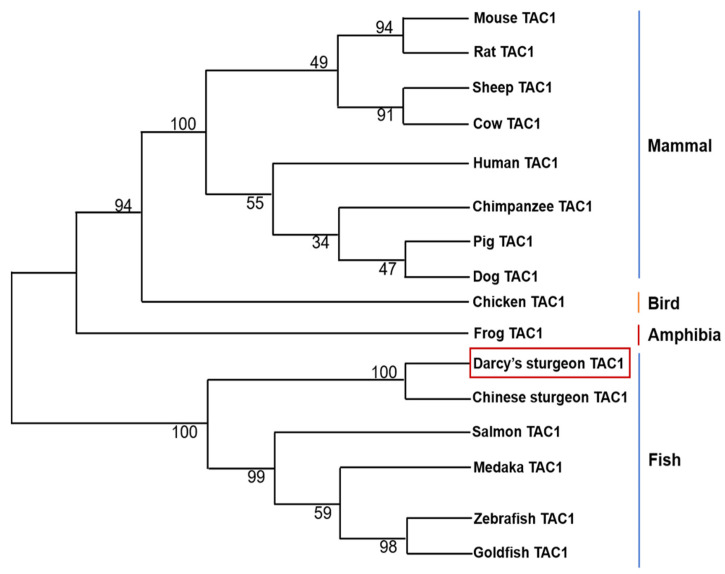
Phylogenetic tree analysis of Dabry’s sturgeon *tac1*.

**Figure 4:**
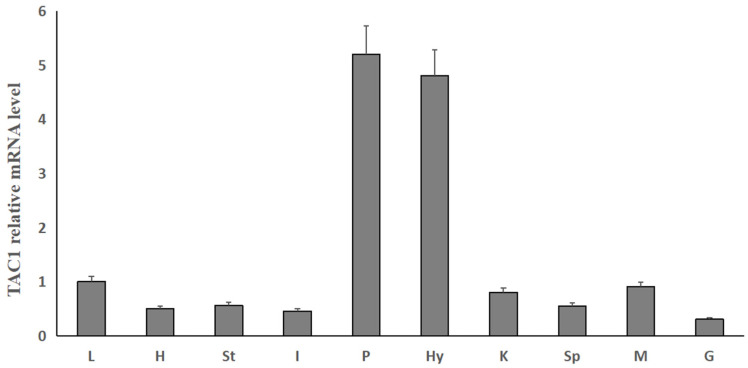
Tissue expression of *tac1* in Dabry’s sturgeon. Notes: L indicates liver, H indicates heart, St indicates stomach, I indicates intestines, P indicates pituitary, Hy indicates hypothalamus, K indicates kidney, Sp indicates spleen, M indicates muscle, and G indicates gonad. Data presented are expressed as mean ± SEM (*n* = 4).

To investigate the structure and tissue expression of *tac1* gene of Dabry’s sturgeon, we first found *tac1* gene of Dabry’s sturgeon from the NCBI. The Dabry’s sturgeon *tac1* gene has a full-length of 339 bp and encoding a 113 a.a. protein precursor. The Dabry’s sturgeon *tac1* gene encodes two mature peptides, namely SP (11 a.a.) and NKA (10 a.a.), which have the same protein cleavage sites (GKR) and have the same sequence “FXGLM” in the C-terminal (Figure 1). The similarity of Tac1 amino acid sequence between Dabry’s sturgeon and Chinese sturgeon, zebrafish, frog, chicken, mouse, and human were 97.3%, 80.0%, 45.0%, 42.0%, 50.0% and 52.3%, respectively (Figure 2). Phylogenetic analysis based on nucleotide sequences further confirms that the newly cloned *tac1* cDNA can be clustered in the clade of fish *tac1* and is closely related to *tac1* in Chinese sturgeon and zebrafish (Figure 3). Tissue expression studies revealed that the newly cloned *tac1* were mainly distributed in the hypothalamus and pituitary (Figure 4).

### 3.2. In Vivo Effect of NKA and SP on Gonadotropin Expression in Dabry’s Sturgeon

**Figure 5:**
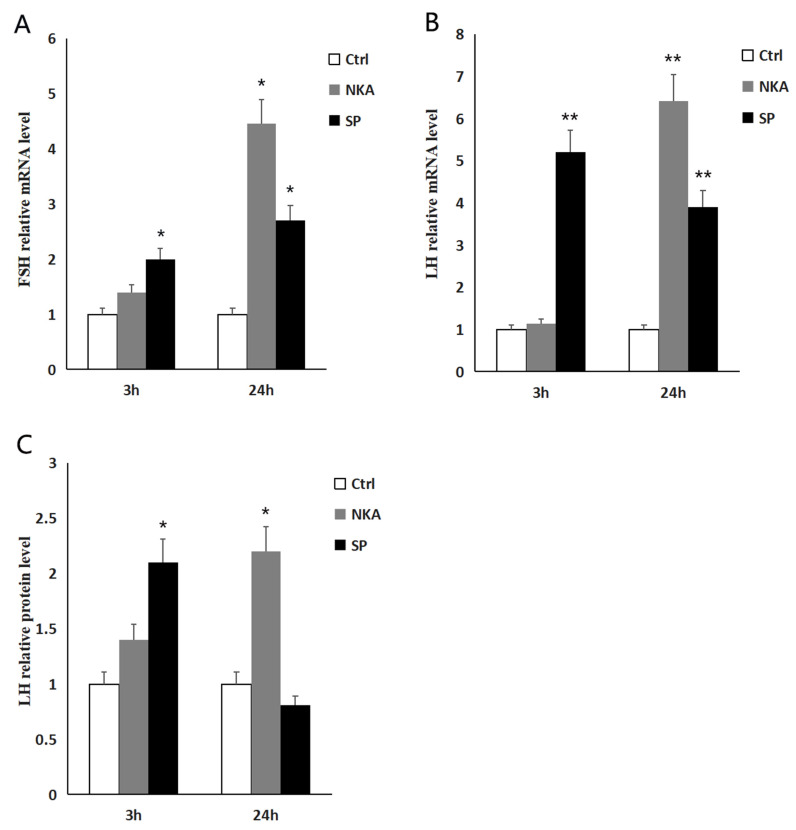
In vivo effect of NKA and SP on *fsh* and *lh* expression in Dabry’s sturgeon. (**A**) The relative *fsh* mRNA level after NKA and SP injection at 3 h and 24 h; (**B**) the relative *lh* mRNA level after NKA and SP injection at 3 h and 24 h; (**C**) the relative content of Lh protein in serum after NKA and SP injection at 3 h and 24 h; data presented are expressed as mean ± SEM (*n* = 4). *p* < 0.05 (“*”) and *p* < 0.01 (“**”) are used to present significant differences among each group. The different letters represent a significant difference at *p* < 0.05 between groups (ANOVA followed by a Dunnett test).

To further clarify the effects of two products of *tac1* gene, NKA and SP, on the pituitary tissue in Dabry’s sturgeon. We used synthetic NKA and SP peptides to intraperitoneally inject Dabry’s sturgeon, and detected the two gonadotropins’ mRNA level in pituitary tissues at 3 h and 24 h after injection, respectively. The Lh protein level in serum was also detected. The two gonadotropins’ mRNA level was detected by real-time quantitative PCR after intraperitoneal injection of synthetic NKA and SP peptides. The relative mRNA level of two gonadotropins at 3 h and 24 h after intraperitoneal injection with NKA and SP are shown in Figure 5A,B. The Lh protein content in serum was detected by ELISA, and the relative protein level of Lh in serum at 3 h and 24 h after intraperitoneal injection with NKA and SP are shown in Figure 5C. The results showed that injection of NKA and SP could promote the two gonadotropins’ mRNA expression in Dabry’s sturgeon in vivo, and also cause the Lh protein content increase in serum.

### 3.3. Effects of TAC1 Gene Products on Gonadotropin in the Pituitary Cells

**Figure 6:**
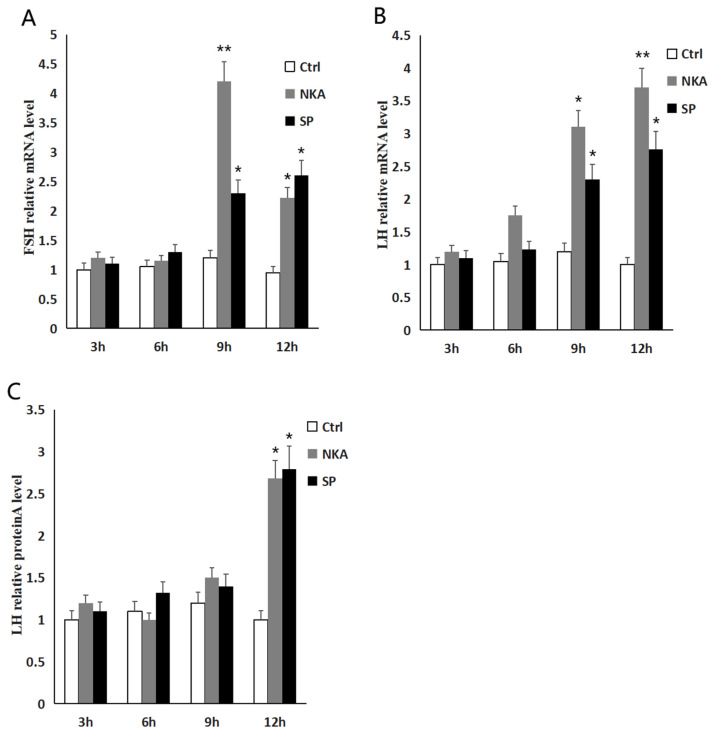
The temporal effects of NKA and SP on *fsh* and *lh* expression in the pituitary cells of Dabry’s sturgeon. (**A**) Relative *fsh* mRNA level in pituitary cells incubated with NKA and SP at different time points; (**B**) relative *lh* mRNA level in pituitary cells incubated with NKA and SP at different time points; (**C**) relative Lh protein content in pituitary cells incubated with NKA and SP at different time points; data presented are expressed as mean ± SEM (*n* = 4). *p* < 0.05 (“*”) and *p* < 0.01 (“**”) are used to present significant differences among each group. The different letters represent a significant difference at *p* < 0.05 between groups (ANOVA followed by a Dunnett test).

In order to verify whether the *tac1* gene product can directly act on the pituitary cells of Dabry’s sturgeon, we extracted the primary pituitary cells. In addition, the primary pituitary cells were treated with different doses of NKA and SP, respectively, and the gene expression of pituitary cells was detected at different time points, so as to explore the time and dose effects of NKA and SP on the primary pituitary cells. The temporal effects of *tac1* gene products on gonadotropin gene expression in the pituitary cells is shown in Figure 6A–C. The regulation of NKA and SP on the secretion of Lh in pituitary cells increased gradually with time, and the maximum secretion was observed at 24 h. The comprehensive results of the effects of different concentrations of NKA and SP on the secretion of Lh in pituitary cells and the relative expression levels of *lh* and *fsh* genes in pituitary cells showed that the maximum effect time of NKA and SP on the regulation of two gonadotropins expression in the pituitary cells was 24 h.

**Figure 7:**
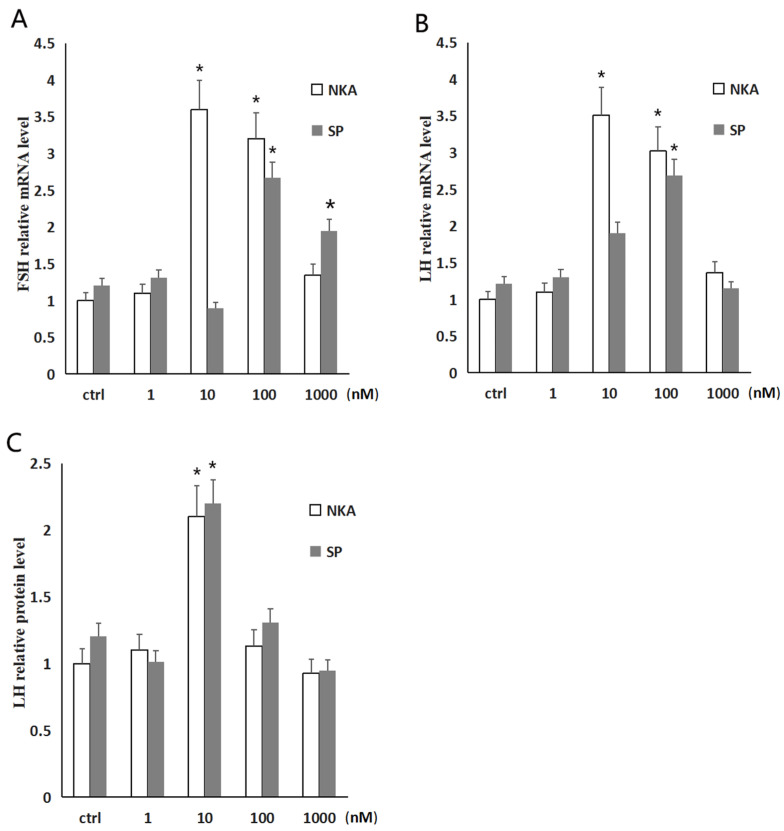
The dose effects of NKA and SP on *fsh* and *lh* expression in the pituitary cells of Dabry’s sturgeon. (**A**) Relative *fsh* mRNA level in pituitary cells incubated with different concentrations of NKA and SP for 24 h; (**B**) relative *lh* mRNA level in pituitary cells incubated with different concentrations of NKA and SP for 24 h; (**C**) relative Lh protein content in pituitary cells incubated with different concentrations of NKA and SP for 24 h; data presented are expressed as mean ± SEM (*n* = 4). *p* < 0.05 (“*”). The different letters represent a significant difference at *p* < 0.05 between groups (ANOVA followed by a Dunnett test).

The dose effects of NKA and SP on gonadotropin gene expression in the pituitary cells is shown in Figure 7A–C. With the increase in NKA and SP treatment concentration, the secretion of Lh in pituitary cells increased first and then decreased, and the maximum treatment concentration of Lh secretion was 10 nM. Among the effects of *tac1* gene products on the two gonadotropins’ genes expression in pituitary cells, the maximum concentration of *lh* relative expression in NKA treated sturgeon cells was 10 nM, and the maximum concentration of *lh* mRNA relative expression in SP treated sturgeon cells was 100 nM. The maximum relative expression concentration of *fsh* mRNA in the pituitary cells treated with NKA was 10 nM, and that in the pituitary cells treated with SP was 100 nM. According to the comprehensive results of the effects of different concentrations of NKA and SP on the secretion of Lh in pituitary cells and the relative expression levels of *lh* and *fsh* genes in the pituitary cells, the maximum effect concentration of NKA and SP on the regulation of the two gonadotropins expression in the pituitary cells was 10 nM.

## 4. Discussion

In mammals, *Tac3* gene product NKB regulates reproductive function mainly by regulating GnRH release in the hypothalamus [23,24]. In rats, the receptor of NKB, NK3R, is expressed on gonadotropin-releasing hormone (GnRH) neurons, and intraventricular injection of NK3R agonists significantly stimulates the release of LH [26]. However, the function of *TAC1* encoding products NKA and SP is still controversial. In ovariectomized rats, when injecting the SP could significantly stimulated the release of LH, while the plasma LH and FSH levels decreased after injected SP antiserum [27]. Similarly, following injections of an anti-NKA serum to rats on diestrus, the prolactin surge during the afternoon of proestrus (PRL) was significantly reduced [28]. These studies suggest that SP and NKA can significantly promote sexual maturation in mammals. However, Battmann et al. [29] found that injecting 100 μg SP into female rats on the day of estrus could significantly reduce the surge of LH before ovulation. At the same time, the results of in vitro experiments also showed that SP treatment significantly reduced the effect of progesterone on GnRH-induced LH release, indicating that SP also had a negative regulatory effect on the reproductive process.

Compared with mammals, there are few reports about *tac1* gene in fish. Early studies have found that SP injection could enhance the learning ability of goldfish through dopamine mediation [30]. Another research showed that the SP/NK1R system is involved in osteogenesis and sexually differential localized expression of *tac1* in the brain in zebrafish [31,32]. Recent studies have found that SP and NKA can promote the Lh and Prl secretion through NK1R and NK2R at the grass carp pituitary cells, which proves that *tac1* encoding products are involved in the regulation of reproductive function in fish [33]. In order to understand the role of SP and NKA in the reproductive process of Dabry’s sturgeon, we cloned *tac1* and analyzed its protein sequence. Our results suggest that the prohormones of the Dabry’s sturgeon can be processed through single/double cleavage sites to produce mature peptides SP and NKA. These peptides share a high degree of homology with peptides from other fish, such as goldfish and zebrafish. In Dabry’s sturgeon, we observed widespread expression of *tac1* in different brain regions at the tissue level, which is consistent with its role as a neurotransmitter. Notably, our study found that *tac1* gene of Dabry’s sturgeon has a high expression level in the pituitary tissue, which is consistent with previous findings in goldfish [34] and rats [35]. This may suggest that *tac1* may also play a functional role in the hypothalamic–pituitary axis of the Dabry’s sturgeon.

In general, neuropeptides act on the hypothalamic–pituitary-gonadal axis mainly through act on the hypothalamus. For example, the kisspeptin could stimulate the expression of GnRH in the hypothalamus to further promote the expression of gonadotropin in the pituitary [36]. However, it has also been reported that neuropeptides can act directly on pituitary cells. SP can directly act on pituitary cells in pigs to induce LH secretion [16]. Similarly, NKA can also induce LH release in vivo of rats, but whether it can direct effect on the pituitary has not been confirmed [15]. In this study, NKA and SP were injected intraperitoneally to detect the gene expression of pituitary tissues of Dabry’s sturgeon, and at the same time, different concentrations of NKA and SP were co-cultured with the pituitary cells. We preliminarily confirmed that the two products of *tac1* gene, NKA and SP, can directly act on the pituitary cells and promote the expression of Fsh and Lh at certain concentrations. This result is consistent with the results of the study on grass carp [32]. Meanwhile, the study on grass carp further proved that NKA and SP regulate the expression of gonadotropin by acting on their downstream receptors NK1R and NK2R, thereby activating downstream signaling pathways. Although our study has not yet proved the existence of SP and NKA receptors in the Dabry’s sturgeon pituitary cells, this issue is worthy of further investigation. In addition, different concentrations of NKA and SP also regulate the expression of Lh and other genes differently. Fergani et al. [37] demonstrated the minimum effective dose of three tachykinins, NKA, SP, and NKB, in sheep. It was found that both NKA (2 nmol) or SP (10 nmol) and low concentrations of NKB (0.2 nmol) stimulated the same level of Lh secretion. Our study also found that NKA and SP had different effects on the expression of gonadotropin in pituitary cells of Dabry’s sturgeon at different doses and time. In this study, we found the concentration of 10 nM, NKA, and SP had the strongest effects on Dabry’s sturgeon primary pituitary cells.

## 5. Conclusions

In conclusion, we conducted biological analysis of *tac1* gene of Dabry’s sturgeon, and found that the Tac1 amino acid sequence of Dabry’s sturgeon was most similar to that of Chinese sturgeon. After tissue expression study, it was found that *tac1* gene was mainly expressed in hypothalamus and pituitary tissue. The core polypeptides of *tac1* gene products NKA and SP were synthesized, and the effect of NKA and SP on the gene expression in pituitary tissue of Dabry’s sturgeon was detected after intraperitoneal injection. The results suggested that the two gonadotropins mRNA level was significantly increased in pituitary tissue and the Lh protein was also increased in serum compared with the control. By culturing primary pituitary cells and co-culture with NKA and SP, our study further demonstrated that NKA and SP can directly act on pituitary cells, and that the optimal dose and time for NKA and SP to act on pituitary cells are 10 nM and 24 h, respectively. Our results preliminarily demonstrated that *tac1* gene products NKA and SP can regulate gene expression by directly acting on pituitary cells of Dabry’s sturgeon. However, the specific molecular mechanism remains to be further studied.

## Figures and Tables

**Table 1:** The primers used in this study.

Primer	Sequence(5′-3′)	Primer Length (bp)	Amplicon Length (bp)
*fsh*-F	CTTACGCAGGCCGATGTG	18	208
*fsh*-R	AGGGTCCAAGGCTTAGGG	18	
*lh*-F	AGGAGGAATGTCCAAAGTGC	20	194
*lh*-R	CAGCGGGAAGGTGAAGTG	18	
*actin*-F	CCTTCTTGGGTATGGAATCTTGC	23	201
*actin*-R	CAGAGTATTTACGCTCAGGTGGG	23	
*fsh*-CDS-F	TATAAAGACATGGCATCAG	19	525
*fsh*-CDS-R	AAGGGAAAGGTTCTAAAC	18	
*lh*-CDS-F	CGACGGCGGGTAAAGGAA	18	491
*lh*-CDS-R	AGCGGGAAGGTGAAGTGGG	19	

## Data Availability

The data presented in this study are available on request from the corresponding author.

## Supplementary Materials

The following supporting information can be downloaded at: https://www.mdpi.com/article/10.3390/ani14020227/s1, Figure S1: The Lh antibody specific detection of Chinese sturgeon and Dabry’s sturgeon by Western blot.

## References

[1] Zhuang P., Ke F., Wei Q., He X., Cen Y. (1997). Biology and life history of Dabry’s sturgeon, *Acipenser dabryanus*, in the Yangtze River. Environ. Biol. Fish..

[2] Zhang H., Wei Q.W., Du H., Li L.X. (2011). Present status and risk for extinction of the Dabry’s sturgeon (*Acipenser dabryanus*) in the Yangtze River watershed: A concern for intensified rehabilitation needs. J. Appl. Ichthyol..

[3] Wei Q.W., Ke F., Zhang J., Zhuang P., Lu J.D., Zhou R.Q., Yang W.H. (1997). Biology, fisheries, and conservation of sturgeons and paddlefish in China. Environ. Biol. Fish..

[4] Wang J.H., Wei Q.W., Zou Y.C. (2011). Conservation strategies for the Chinese sturgeon, *Acipenser sinesis*: An overview on 30 years of practices and future needs. J. Appl. Ichthyol..

[5] Zohar Y., Muñoz-Cueto J.A., Elizur A., Kah O. (2010). Neuroendocrinology of reproduction in teleost fish. Gen. Comp. Endocr..

[6] Corander M.P., Challis B.G., Thompson E.L., Jovanovic Z., Loraine Tung Y.C., Rimmington D., Huhtaniemi I.T., Murphy K.G., Topaloglu A.K., Yeo G.S. (2010). The effects of neurokinin B upon gonadotrophin release in male rodents. J. Neuroendocrinol..

[7] Almeida T.A., Rojo J., Nieto P.M., Pinto F.M., Hernandez M., Martin J.D., Candenas M.L. (2004). Tachykinins and tachykinin receptors: Structure and activity relationships. Curr. Med. Chem..

[8] Steinhoff M.S., von Mentzer B., Geppetti P., Pothoulakis C., Bunnett N.W. (2014). Tachykinins and their receptors: Contributions to physiological control and the mechanisms of disease. Physiol. Rev..

[9] Pennefather J.N., Lecci A., Candenas M.L., Patak E., Pinto F.M., Maggi C.A. (2004). Tachykinins and tachykinin receptors: A growing family. Life. Sci..

[10] Lin C.C., Chen W.N., Chen C.J., Lin Y.W., Zimmer A., Chen C.C. (2012). An antinociceptive role for Substance P in acid-induced chronic muscle pain. Proc. Natl. Acad. Sci. USA.

[11] Ang S.F., Moochhala S.M., MacAry P.A., Bhatia M. (2011). Hydrogen sulfide and neurogenic inflammation in polymicrobial sepsis: Involvement of Substance P and ERK-NF-κB signaling. PLoS ONE.

[12] Cipriani G., Serboiu C.S., Gherghiceanu M., Faussone-Pellegrini M.S., Vannucchi M.G. (2011). NK receptors, Substance P, Ano1 expression and ultrastructural features of the muscle coat in Cav-1^−/−^ mouse ileum. J. Cell. Mol. Med..

[13] Shaffer A.D., Ball C.L., Robbins M.T., Ness T.J., Randich A. (2011). Effects of acute adult and early-in-life bladder inflammation on bladder neuropeptides in adult female rats. BMC. Urol..

[14] Trivedi C., Shan X., Tung Y.C., Kabra D., Holland J., Amburgy S., Heppner K., Kirchner H., Yeo G.S., Perez-Tilve D. (2015). Tachykinin-1 in the central nervous system regulates adiposity in rodents. Endocrinology.

[15] Debeljuk L., Lasaga M. (1999). Modulation of the hypothalamo-pituitary-gonadal axis and the pineal gland by neurokinin A, neuropeptide K and neuropeptide gamma. Peptides.

[16] Hidalgo-Diaz C., Castano J.P., Lopez-Pedrera R., Malagon M.M., Garcia-Navarro S., Gracia-Navarro F. (1998). A modulatory role for substance P on the regulation of luteinizing hormone secretion by cultured porcine gonadotrophs. Biol. Reprod..

[17] Biran J., Palevitch O., Ben-Dor S., Levavi-Sivan B. (2012). Neurokinin Bs and neurokinin B receptors in zebrafish–potential role in controlling fish reproduction. Proc. Natl. Acad. Sci. USA.

[18] Zhou W., Li S., Liu Y., Qi X., Chen H., Cheng C.H., Liu X., Zhang Y., Lin H.R. (2012). The evolution of tachykinin/tachykinin receptor (*TAC/TACR*) in vertebrates and molecular identification of the *TAC3/TACR3* system in zebrafish (*Danio rerio*). Mol. Cell. Endocrinol..

[19] Biran J., Golan M., Mizrahi N., Ogawa S., Parhar I.S., Levavi-Sivan B. (2014). Direct regulation of gonadotropin release by neurokinin B in tilapia (*Oreochromis niloticus*). Endocrinology.

[20] Qi X., Zhou W.Y., Li S.S., Liu Y., Ye G., Liu X.C., Peng C., Zhang Y., Lin H.R. (2015). Goldfish neurokinin B: Cloning, tissue distribution, and potential role in regulating reproduction. Gen. Comp. Endocrinol..

[21] Lin C.Y., Jiang X., Hu G.F., Ko K.W., Wong O.L. (2015). Grass carp prolactin: Molecular cloning, tissue expression, intrapituitary autoregulation by prolactin and paracrine regulation by growth hormone and luteinizing hormone. Mol. Cell. Enodcrinol..

[22] Hu G.F., He M.L., Ko W.K., Lin C.Y., Wong A.O. (2014). Novel pituitary actions of TAC3 gene products in fish model: Receptor specificity and signal transduction for prolactin and somatolactin alpha regulation by neurokinin B (NKB) and NKB-related peptide in carp pituitary cells. Endocrinology.

[23] Lopez-Bellido R., Barreto-Valer K., Rodriguez R.E. (2013). Substance P mRNA expression during zebrafish development: Influence of mu opioid receptor and cocaine. Neuroscience.

[24] Hu G.F., He M.L., Ko W.K., Wong A.O. (2017). *TAC1* gene products regulate pituitary hormone secretion and gene expression in prepubertal grass carp pituitary cells. Endocrinology.

[25] Hu Q.Y., Qin Q.B., Xu S.H., Zhou L.L., Xia C.H., Shi X.T., Zhang H.Y., Jia J.Y., Yin Z., Hu G.H. (2020). Pituitary actions of EGF on gonadotropins, growth hormone, prolactin and somatolactins in grass carp. Biology.

[26] Sandoval-Guzmán T., Rance N.E. (2004). Central injection of senktide, an NK3 receptor agonist, or neuropeptide Y inhibits LH secretion and induces different patterns of Fos expression in the rat hypothalamus. Brain. Res..

[27] Arisawa M., De Palatis L., Ho R., Snyder G.D., Yu W.H., Pan G., McCann S.M. (1990). Stimulatory role of substance P on gonadotropin release in ovariectomized rats. Neuroendocrinology.

[28] Pisera D., Debeljuk L., Seilicovich A., Afione S., Duvilanski B., Diaz M.C., Lasaga M., Traktemberg R., Bartke A. (1991). Possible role of neurokinin A in the control of prolactin secretion in rats and hamsters. J. Neuroendocrinol..

[29] Battmann T., Mĕlik Parsadaniantz S., Jeanjean B., Kerdelhué B. (1991). In-vivo inhibition of the preovulatory LH surge by substance P and in-vitro modulation of gonadotrophin-releasing hormone-induced LH release by substance P, oestradiol and progesterone in the female rat. J. Endocrinol..

[30] Mattioli R., Santangelo E.M., Costa A.C., Vasconcelos L. (1997). Substance P facilitates memory in goldfish in an appetitively motivated learning task. Behav. Brain. Res..

[31] Ytteborg E., Torgersen J.S., Pedersen M.E., Helland S.J., Grisdale-Helland B., Takle H. (2013). Exercise induced mechano-sensing and substance P mediated bone modeling in Atlantic salmon. Bone.

[32] Ogawa S., Ramadasan P.N., Anthonysamy R., Parhar I.S. (2021). Sexual dimorphic distribution of hypothalamic Tachykinin1 cells and their innervations to GnRH neurons in the zebrafish. Front. Endocrinol..

[33] Hu G.F., Lin C.Y., He M.L., Wong A.O. (2014). Neurokinin B and reproductive functions: “KNDy neuron” model in mammals and the emerging story in fish. Gen. Comp. Endocrinol..

[34] Topaloglu A.K., Reimann F., Guclu M., Yalin A.S., Kotan L.D., Porter K.M., Serin A., Mungan N.O., Cook J.R., Ozbek M.N. (2009). *TAC3* and *TACR3* mutations in familial hypogonadotropic hypogonadism reveal a key role for Neurokinin B in the central control of reproduction. Nat. Genet..

[35] Ogawa S., Ramadasan P.N., Goschorska M., Anantharajah A., Ng K.W., Parhar I.S. (2012). Cloning and expression of tachykinins and their association with kisspeptins in the brains of zebrafish. J. Comp. Neurol..

[36] Huang H.T., Xiao K., Shu T.T., Liu X.Q., Yang J. (2023). Effects of Kisspeptin on the reproductive function in the Dabry’s sturgeon (*Acipenser dabrynus*). Gen. Comp. Endocrinol..

[37] Fergani C., Mazzella L., Coolen L.M., McCosh R.B., Hardy S.L., Newcomb N., Grachev P., Lehman M.N., Goodman R.L. (2016). Do substance P and neurokinin A play important roles in the control of LH secretion in ewes?. Endocrinology.

