# Supplementary material for: The Effects of Tachykinin1 Gene Products on Prepubertal Dabry’s Sturgeon (Acipenser dabrynus) Pituitary Hormone Secretion and Gene Expression
Source: Animals (Basel). 2024 Jan 11;14(2):227. doi: 10.3390/ani14020227 (PMC10812735; doi:10.3390/ani14020227)
Supplement: Supplementary file 1 [file animals-14-00227-s001.zip › animals-2737134-supplementary.pdf]

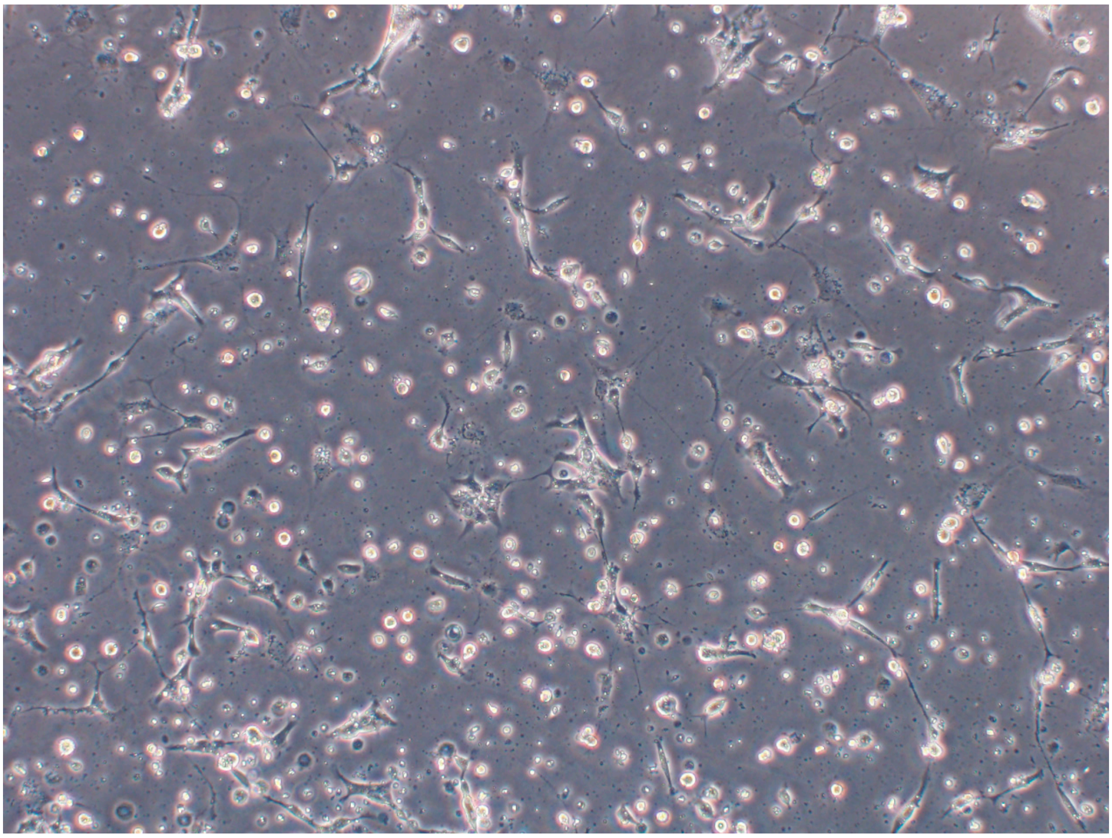

The Primary pituitary cells of Dabry's sturgeon

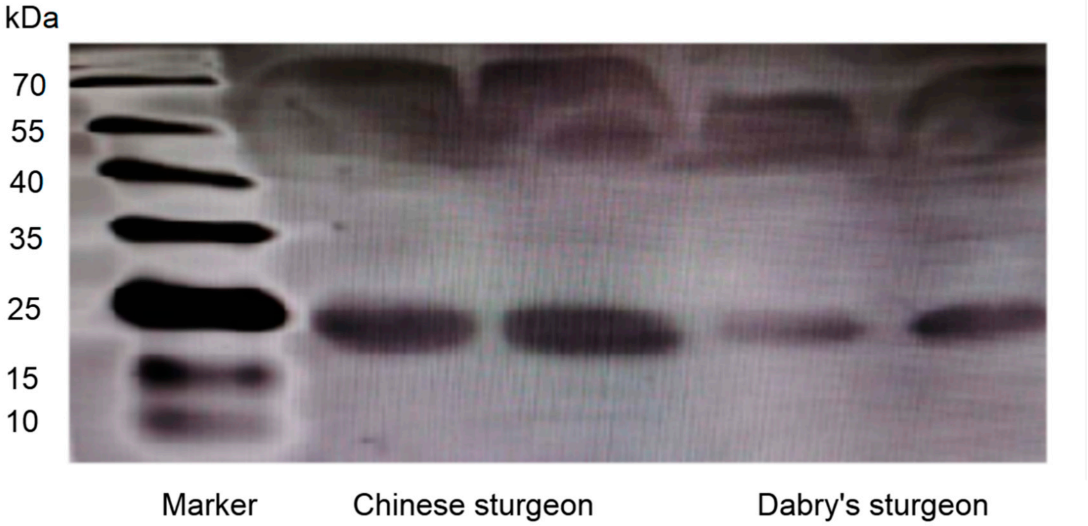

Figure S1. The Lh antibody specific detection of Chinese sturgeon and Dabry's sturgeon by western-blot
